# The disposable male— the ultimate emancipation of females?

**DOI:** 10.1186/s12915-018-0574-8

**Published:** 2018-09-25

**Authors:** Duur K. Aanen

**Affiliations:** Department of Plant Sciences, Laboratory of Genetics, Wageningen University, 6708 PB Wageningen, Wageningen, The Netherlands

## Abstract

Sexual reproduction is costly compared to asexual reproduction, in particular because males generally contribute little to offspring. Research published today in *BMC Biology* shows that some populations of a termite species have disposed of males altogether. However, this need not necessarily be seen as a victory for the females, since males in most termite societies are active colony members that contribute their fair share to colony tasks.

## Commentary

The defining distinction between males and females is based on investment in the zygote: females provide the bulk of the cytoplasm via a large egg, while males contribute virtually nothing with either a tiny sperm cell or pollen grain. In species with internal fertilisation and/or brood care, this asymmetry in investment can extend to later developmental stages of the offspring, since males often contribute little or nothing. John Maynard-Smith argued that this difference in investment implies that sexual reproduction has a twofold cost compared to asexual [1]. In an outcrossing sexual population, the stable sex ratio is 50:50, so on average individuals ‘waste’ 50% of resources on males who inefficiently convert resources into offspring. A mutation that induces asexuality in a sexual population with two separate sexes would initially double in frequency each generation, since, all else being equal, asexuality is exactly twice as efficient at converting resources into descendants.

A new study in *BMC Biology* shows that termite species can abandon males altogether, consistent with Maynard-Smith’s idea that asexual variants can gain a short-term benefit over sexual relatives. Queens of some populations of the species *Glyptotermes nakajimai* reproduce without any genetic contribution by males, not only to produce new queens, but also to produce sterile workers and soldiers [2]. Dissections of more than 4200 individuals, belonging to soldiers, workers and reproductives of 37 colonies from six populations, did not uncover a single male. Colonies of the four other populations of the same species all contained both male and female individuals in roughly equal numbers, for reproductive, worker and soldier castes. In further support of the absence of males, the spermathecae—the storage organs for sperm—of queens of the six all-female populations did not carry any sperm, while spermathecae of queens from the other populations were filled with sperm. The exact mechanism whereby reproduction without fertilization by males occurs is unknown, either by some form of self-fertilisation (automixis, either via gamete duplication or gamete fusion) or purely asexual reproduction (apomixis) where eggs are produced by mitosis. Since the chromosome number of the asexual populations likely is uneven, apomixis is most plausible.

**Fig. 1:**
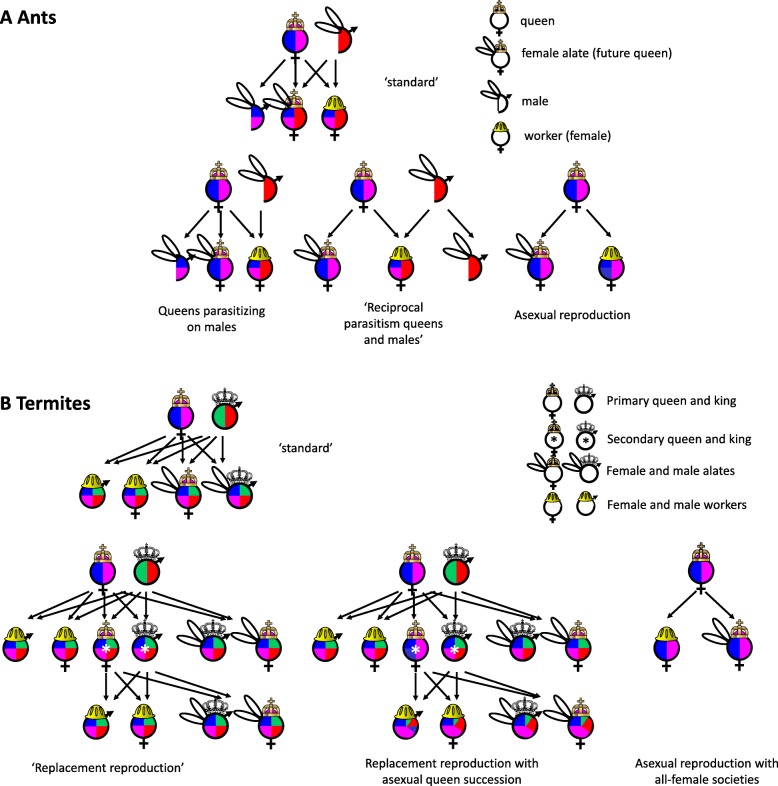
Standard life cycles of ants (**a**) and termites (**b**) and deviations due to conflicts between the reproductive interests of queens and males. The genome representation of ancestors is indicated in colours in the offspring (adapted with permission from a sketch made by David Nash). **a** Ants have a haplo-diploid life cycle, where unfertilized eggs become males and fertilized eggs either workers or female alates. All workers in an ant society are female. In some ants, queens reproduce parthenogenetically to produce alates, but sexually to produce workers, thus parasitizing on males [5]. In yet another deviation, queens and males each produce their own female and male alates via asexual reproduction, but workers via sexual reproduction [6]. Finally, some ant species have become obligately asexual, where both workers and new queens are produced without any contribution of males [7]. **b** In contrast to ants, termites are diploid social insects. In the ‘standard’ life cycle, a single queen and king found a colony and produce sterile helpers (workers and soldiers) and fertile alates via sexual reproduction. In some species, replacement reproduction occurs, where the primary queen and king can be replaced by their own offspring [8]. In some species the queen can produce a replacement queen by asexual reproduction [9]. Finally, some populations of the species *Glyptotermes nakajimai* have all-female societies, which form alates and workers via asexual reproduction [2]

By producing all-female societies, colonies of this termite resemble colonies of Hymenopteran social insects, the ants, social wasps and bees, where sterile helpers are exclusively female. It was long believed that this sex bias is a consequence of their peculiar sex determination. Sex in Hymenoptera is determined by ploidy: unfertilized, haploid, eggs develop into males, while fertilized, diploid, eggs develop into females. Haplo-diploid sex determination has interesting implications for relatedness (*r*) among different colony members (Fig. 1a)*.* In a colony founded by a single queen mated with a single male, daughters have a relatedness of ¾ with their sisters, since they all have half of their genome in common via the haploid father and additionally on average one quarter via their diploid mother. In contrast, their relatedness to their own offspring is only ½. According to the haplo-diploidy hypothesis, proposed by Bill Hamilton, this difference in relatedness would predispose females to become workers, as they could increase their inclusive fitness more by helping their mother raise sisters to become new queens than by producing new queens themselves [3].

However, the flipside of haplo-diploid sex determination is that sisters are less related to their brothers (*r* = ¼) than to their own sons (*r* = ½). Later on it was realised that this implies that all-female societies of Hymenoptera cannot be explained by the haplo-diploidy hypothesis, since the average relatedness among females and siblings is ½, exactly equal to relatedness with own offspring. Instead, a recent analysis found support for the hypothesis that the sex of helpers can be explained by variation in the ecological factors that favoured eusociality [4]. According to this idea, if the original task of helpers was to rear brood, we would expect the helpers to be drawn from the sex or sexes that provided parental care in the ancestral non-social species, which is usually females. The original task of helpers in social Hymenoptera was indeed brood rearing. In contrast, in termites it is likely that helpers originally had multiple tasks, including colony defence. Since the ancestors of termites occupied wood trunks that provided their food, they lived inside their food, which constituted a valuable resource worth defending against competitors. Since neither sex is pre-adapted for defensive tasks, we would expect the helpers to be drawn from both sexes. This could explain why helper castes in most termite species usually are a mixture of male and female individuals.

Even though high relatedness among sisters is no longer believed to explain the sex of workers of Hymenopteran species, kin-selection theory does provide the explanation for the evolution of the extreme altruism seen in societies of social insects. By helping their mother produce fertile offspring, sterile individuals can increase their inclusive fitness via the genes present in genetically related individuals. However, differences in relatedness between colony members also provide a rich ground for conflicts between different colony members. Kin conflicts have been studied most extensively in ants (Fig. 1a). Some remarkable outcomes of such conflicts have been described recently. In the ant species *Cataglyphis cursor*, males and workers are produced via normal sexual reproduction, from unfertilized and fertilized eggs, respectively, but queens clone themselves to produce new queens [5]. Queens thus parasitize on males, since males do not contribute any genetic material to reproductives of the next generation, but only to workers. In other cases, however, the conflict between males and females over transmitting genes has resulted in a draw. In a few ant species, workers are produced sexually, but female and male reproductives asexually. In those cases, it is thought that the males manage to exclude the maternal genome from fertilised eggs, thus clonally propagating themselves [6]. Interestingly, in those cases, males and females represent evolutionarily completely separated lineages whose genomes only come together in the workers. Finally, like the newly discovered termite populations, some ant species have also disposed of males completely, and reproduce asexually [7].

In termites, kin conflicts have been studied less extensively (Fig. 1b). In some species, one or more offspring can replace a primary reproductive that has died and become a replacement or secondary reproductive. Replacement reproduction results in inbreeding and can happen repeatedly in a single colony. This was once believed to be important for the evolution of reproductive altruism in diploid organisms, since inbreeding increases relatedness among colony members [8]. In 2009, Matsuura and co-workers discovered that in some termite species, secondary queens are produced by parthenogenesis of the primary queen, so-called asexual queen succession (AQS) [9]. By cloning herself, the queen can extend her (genetic) lifespan. An additional consequence is that the queen can increase her genetic contribution to offspring beyond the 50% that results from normal reproduction. The reason is that the primary king cannot produce secondary kings by parthenogenetic reproduction, but only by mating with the queen.

**Fig. 2:**
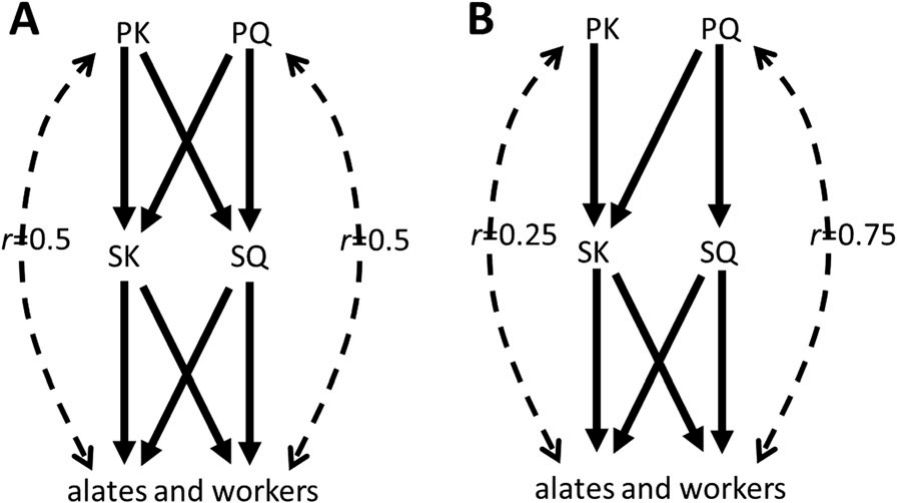
The consequences of replacement reproduction for relatedness of colony members to the primary queen and king. **a** ‘Standard’ replacement reproduction, where secondary reproductives are both produced sexually by the primary reproductives. **b** Replacement reproduction where the secondary queen is formed by asexual queen succession, so that the primary queen extends her genetic life span, but the secondary king is formed by sexual reproduction of the primary king and queen

The one-sided, mother–son inbreeding which can result from mating between the cloned queen and the secondary king in long-lived colonies implies that colony members are then related by ¾ to the primary queen, and only by ¼ to the primary king [10] (Fig. 2). This implies that, from an inclusive-fitness perspective, female reproductives are more valuable for colony members, both male and female, than male reproductives. Kin-selection theory therefore predicts that workers should favour a female-biased sex ratio of the alates, which is supported by empirical evidence for several species with AQS, and, as expected, not for species without AQS [9]. Here, I want to propose another possible corollary of this difference in relatedness between helpers and the primary king and relatedness between helpers and the primary queen. Since sex determination in termites is chromosomally based on an XY system, random segregation of X and Y chromosomes will lead to an equal sex ratio. However, since caste of an offspring is determined by environmental factors influenced by helpers, helpers can influence the sex ratio of the alates. Since helpers, irrespective of their sex, all have an interest in a more female-biased sex ratio of the alates, a direct consequence of raising more female offspring as alates may be that a larger proportion of the remaining offspring that become helpers are male. A testable prediction, therefore, is that species with AQS, in conjunction with a more female-biased sex ratio of the alates, have a more male-biased sex ratio of the helper castes. Consistent with this prediction, the species *Reticulitermes virginicus*, which has AQS and the most strongly female-biased alate sex ratios, shows a significantly male-biased worker sex ratio, although the sex ratio of soldiers is female biased (Matsuura, personal communication). Clearly, more research is needed to test this hypothesis.

The increased tendency for the helper castes to become male biased for species with AQS may constrain the evolution of all-female asexual reproduction of such species, since they rely on males as a work force. It therefore may make sense that the all-female societies of *G. nakajimai* discovered now [2] belong to a family where AQS is not known (Kalotermitidae). It is ironic that in the group where males contribute more to raising the offspring than any other group of social insects, females of some populations have gotten rid of males. Yet, the benefit of saving on the twofold cost of sex remains: since males contribute so little to the zygote, diverting resources away from males towards females can be selected for. In *G. nakajimai* the more uniform morphology of the soldier caste appears to provide an additional advantage in colony defence, allowing asexual populations to maintain a smaller soldier force [2].

The rarity of purely asexual reproduction and the empirical finding that asexual lineages generally represent terminal branches in the tree of life suggest that asexuality is doomed to death on the long term. Nevertheless, the split between the sampled asexual and sexual lineages in *Glyptotermes nakajimai* is estimated to have occurred 14 million years ago [2]. However, as acknowledged by the authors, this time estimate may not indicate the split of asexuality from sexual ancestors, but only the maximum divergence time. Finding populations intermediate in divergence from those two clusters of lineages could give a more realistic estimation of the age of asexuality in this termite species. Time will thus have to tell how long these colonies of emancipated females have managed to survive without males.
